# To bead or not to bead: A review of *Pseudomonas aeruginosa* lung infection models for cystic fibrosis

**DOI:** 10.3389/fphys.2023.1104856

**Published:** 2023-02-07

**Authors:** Nicole Reyne, Alexandra McCarron, Patricia Cmielewski, David Parsons, Martin Donnelley

**Affiliations:** ^1^ Robinson Research Institute, University of Adelaide, Adelaide, SA, Australia; ^2^ Adelaide Medical School, University of Adelaide, Adelaide, SA, Australia; ^3^ Respiratory and Sleep Medicine, Women’s and Children’s Hospital, North Adelaide, SA, Australia

**Keywords:** *Pseudomonas aeruginosa*, animal models, lung disease, cystic fibrosis, lung infection

## Abstract

Cystic fibrosis (CF) lung disease is characterised by recurring bacterial infections resulting in inflammation, lung damage and ultimately respiratory failure. *Pseudomonas aeruginosa* is considered one of the most important lung pathogens in those with cystic fibrosis. While multiple cystic fibrosis animal models have been developed, many fail to mirror the cystic fibrosis lung disease of humans, including the colonisation by opportunistic environmental pathogens. Delivering bacteria to the lungs of animals in different forms is a way to model cystic fibrosis bacterial lung infections and disease. This review presents an overview of previous models, and factors to consider when generating a new *P. aeruginosa* lung infection model. The future development and application of lung infection models that more accurately reflect human cystic fibrosis lung disease has the potential to assist in understanding the pathophysiology of cystic fibrosis lung disease and for developing treatments.

## Cystic fibrosis lung disease

Cystic fibrosis (CF) is a genetic disorder that arises due to mutations in the CF transmembrane conductance regulator (*CFTR*) gene, a chloride and bicarbonate ion transport channel that contributes to the absorption and secretion of ions across epithelial surfaces in the body (Malhotra et al., 2019). CF affects multiple organs including the pancreas, digestive system, reproductive system and sweat glands, however it is often the respiratory system that displays the most severe pathological consequences. *CFTR* mutations results in an absence of chloride transport and hyperabsorption of sodium ions. This ion imbalance leads to water absorption into the tissue, dehydration of the airway surface liquid (ASL), accumulation of abnormal sticky mucus, and subsequent impairment of the mucociliary clearance (Sousa and Pereira, 2014). Opportunistic inhaled bacteria adhere to the mucus and are unable to be cleared, resulting in inflammation and a predisposition to acute and ultimately chronic lung infections (Malhotra et al., 2019). In people with CF these infections result in progressive lung damage throughout the lifetime, which eventually causes respiratory failure.

### *Pseudomonas aeruginosa* adaptation to cystic fibrosis lung environment

Initial lung infections with common CF pathogens primarily arises from environmental inhalation (Ramsay et al., 2016). CF lungs are typically infected with multiple bacterial species that change in prevalence with age. Shortly after birth initial airway infections are most frequently caused by *Staphylococcus aureus* and *Haemophilus influenzae*. In contrast *P. aeruginosa* is the dominant bacteria in older children and adults, with 80% of people with CF succumbing to respiratory failure brought on by infection with this bacterial species (Bhagirath et al., 2016). Other notable organisms involved in CF lung infections include *Burkholderia cepacia, Stenotrophomonas maltophilia* and *Achromobacter xylosoxidans* (Folkesson et al., 2012).

*Pseudomonas aeruginosa* is an aerobic Gram-negative rod-shaped bacterium with a widespread presence in the environment. While it may be occasionally transmitted to humans without CF as an opportunistic infection, resulting in pneumonia, wound infections and/or urinary tract infections, it rarely infects healthy lungs (Hogardt and Heesemann, 2010). As people with CF have poor lung defence mechanisms, they are highly susceptible to infections by *P. aeruginosa*. Initial infections may be combated with aggressive antibiotic therapy, delaying the establishment of *P. aeruginosa* until adulthood (Döring et al., 2012). However, once *P. aeruginosa* colonises the CF airways it is very challenging to eradicate, with 60%–80% of CF adults showing chronic infection by this pathogen (Parkins et al., 2018). CF children that are colonised with *P. aeruginosa* infection early in childhood have a poorer prognosis and life expectancy, compared to those that become infected later in life (Stuart et al., 2010).

*Pseudomonas aeruginosa* has a large genome of around 6,000 genes, some of which are associated with metabolic pathways, virulence, and transport (Sousa and Pereira, 2014). The majority of childhood infections occur with unique, non-clonal strains that are obtained from the local environment (Parkins et al., 2018). Older people with CF tend to share strains that have different genetic and phenotypic properties from the environmental *P. aeruginosa* strains. For *P. aeruginosa* to survive and dominate in the CF lung environment it must adapt to stressful conditions arising from bacterial competition, host defense mechanisms, and antibiotic treatment (Bhagirath et al., 2016). Over time selective pressure causes a fundamental transition in the gene expression profile and an adaptation process that is accelerated by mutator phenotypes (Sousa and Pereira, 2014). These changes in gene expression can impart altered characteristics including increased resistance to antibiotics, decreased metabolism, slower growth rate, lack of motility due to loss of flagella and overproduction of alginate (Bhagirath et al., 2016).

**FIGURE 1:**
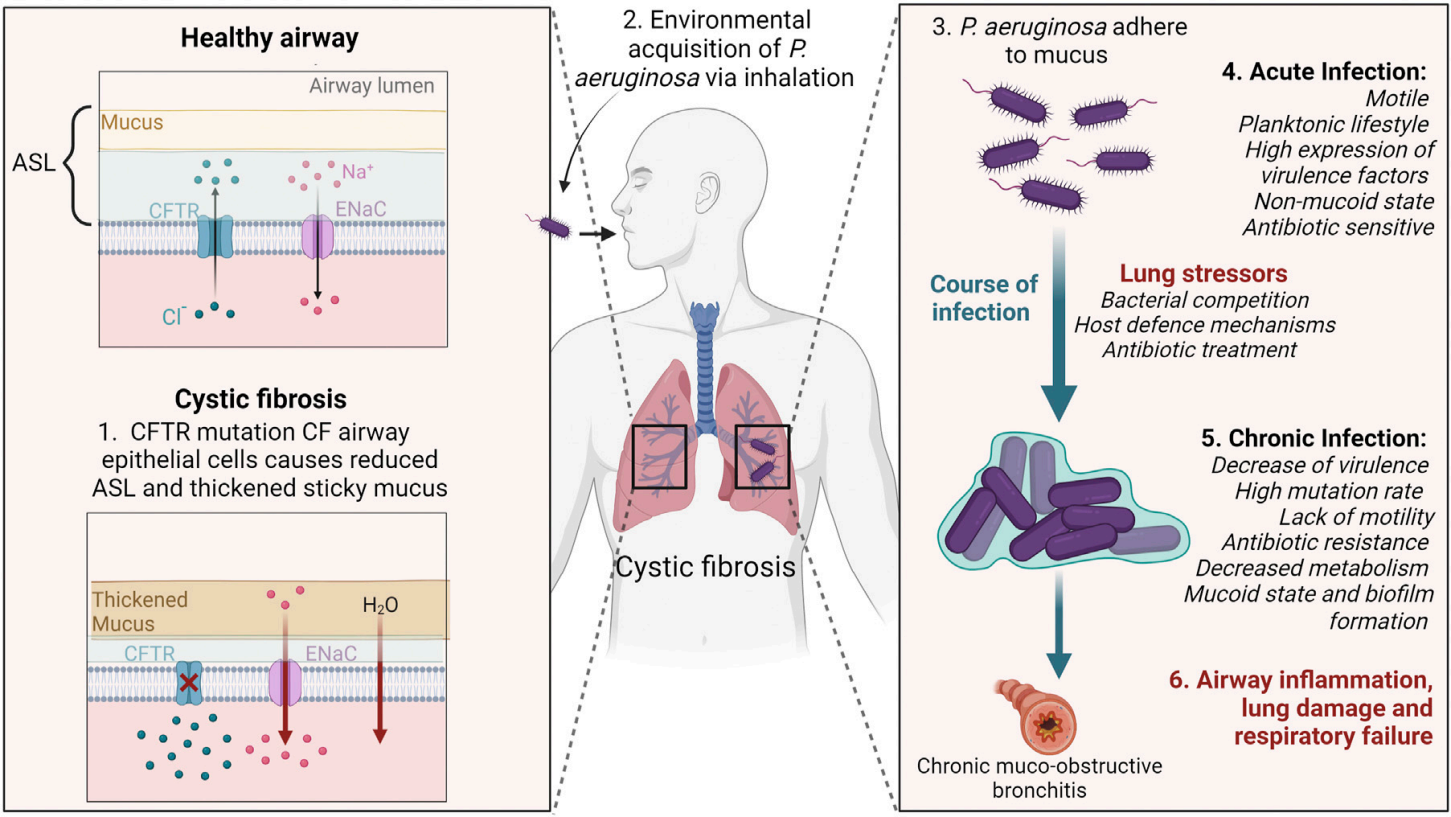
Schematic illustrating *Pseudomonas aeruginosa* infection and adaptation to cystic fibrosis (CF) lungs. CFTR mutations reduce chloride transport in the airways causing hyperabsorption of sodium, and water to be absorbed into the tissue. In turn, the ASL becomes dehydrated, thickened sticky mucus accumulates and mucocillary clearance is impaired. Environmentally acquired *P. aeruginosa* adhere to the mucus and cannot be cleared. During early infection stages *P. aeruginosa* have flagella and pili present for motility. The stressful conditions of the CF lung creates selective pressures that cause *P. aeruginosa* to accumulate gene mutations enabling it to adapt to the lung environment. In the chronic infection stage, *P. aeruginosa* develops characteristics including loss of the flagella and pili, overproduction of alginate biofilm, and resistance to antibiotics. Chronic lung infections result in airway inflammation, lung tissue damage and eventually respiratory failure. CFTR; cystic fibrosis transmembrane conductance regulator, ENaC; epithelial sodium channel, ASL; airway surface liquid. Created with BioRender.

The overproduction of alginate results in formation of thick biofilms and characterises the conversion of non-mucoid *P. aeruginosa* to a mucoid state (Folkesson et al., 2012). These biofilms are made up of micro-colonies of bacteria encased in a polysaccharide matrix, predominantly alginate, creating a physical barrier that protects the bacteria from phagocytosis and antibiotic therapy (Jensen et al., 2007) (Figure 1). The onset of mucoid *P. aeruginosa* is associated with poor prognosis, deterioration of lung function, and increased tissue damage (Hogardt and Heesemann, 2010). Mucoid variants of *P. aeruginosa* are rarely isolated from non-CF environments, thus being unique to CF (Jelsbak et al., 2007; Damkiær et al., 2013).

### Animal models of cystic fibrosis

The importance of CF animal models for understanding disease pathophysiology and for the development of new therapies is well recognised, and these models have previously been reviewed in McCarron et al., 2021 (McCarron et al., 2021). While CF animal models recapitulate varying degrees of CF pathophysiology that include gut obstructions, reproductive defects and nasal bioelectrical defects, many do not exhibit lung disease. Although the CF pig (Stoltz et al., 2010) and ferret (Sun et al., 2014) develop some features of CF lung disease, including infections, access to these models is limited. Specifically, for Australian researchers, import and biosecurity issues prevent their use, whereas financial implications and additional husbandry requirements are a major limiting factor across CF research. Smaller CF animal models, notably mice and rats, do not replicate the spontaneous colonisation with pathogens like *P. aeruginosa*, and that may be in part due to the housing of rodents in specific pathogen free facilities, among other factors (McCarron et al., 2018). Consequently, the lack of access to an animal model with CF-like lung disease limits our further understanding in pathophysiology and treatment studies.

### Animal models of *Pseudomonas aeruginosa* lung infection

Administering bacteria directly into the lungs is a method commonly used to mimic a range of lung infections for CF and other diseases (Evans et al., 2010; McConnell et al., 2010; Bonfield et al., 2021). Various animal species/strains have been used to generate *P. aeruginosa* infection models, including genetically modified species that have *CFTR* mutations. Accordingly, this review is intended to provide an overview of *P. aeruginosa* lung infection models that have been developed to resemble CF lung disease. In particular, the factors that should be considered for establishing these models will be discussed.

#### Choice of *Pseudomonas aeruginosa* strain

Multiple strains of *P. aeruginosa* have been used to develop CF-like lung infections, with laboratory strains and clinical isolates of *P. aeruginosa* having been tested (Table 1). PAO1 is the most common laboratory strain, first isolated from a wound infection in 1954, and has since had its entire genome sequenced (Klockgether et al., 2010). PAO1 is a non-mucoid, motile *P. aeruginosa* strain and is considered more virulent and faster replicating than clinical isolates collected from the lungs of people with CF. While the core genome is conserved across all *P. aeruginosa* strains, clinical isolates demonstrate modifications to the genes responsible for virulence phenotype, mucoidity, mobility, and replication (Finnan et al., 2004; Hoffmann et al., 2005).

**TABLE 1:** Overview of *Pseudomonas aeruginosa* lung infection models. Note that a more comprehensive electronic filterable spreadsheet version is included as supplementary material.

Year	Author	Mode of infection	Planktonic/beads	Species	Strain	Colony forming units (CFU) (µL)	Volume (µL)	Lab or clinical isolate	*Pseudomonas areuginosa* strain	Duration	Ref
1979	Cash	Tracheotomized	Agar beads	Rat	Sprague Dawley	1 × 10^4^	50	Isolate	DGS and DGM (mucoid)	3, 7, 14, 21, 25 days	Cash et al. (1979)
1987	Starke	Tracheotomized	Agar beads	Mouse	Swiss CD1	1 × 10^3^–2 × 10^3^	50	Isolate	PA 2192 (mucoid and non-mucoid)	10 or 21 days	Starke et al. (1987)
2000	van Heeckeren	Tracheotomized	Agar beads	Mouse	C57BL/6	6.5 × 10^4^	50	Isolate	PA M57-15	3 h, 1, 2,3, 4, 7, 10 days	Van Heeckeren et al. (2000)
2001	Schroeder	Intranasal	Planktonic	Mouse	BALB/c, C57BL/6 and CF strains F508 cftr, S489X cftr	1 × 10^7^–2 × 10^7^	10	Both	PA01, 149, 324 (non-mucoid isolates). 6294FRD1 (mucoid isolate)	4.5 h	Schroeder et al. (2001)
		Orotracheal	Planktonic	Mouse			20–50	Both			
2002	Chmiel	Tracheotomized	Planktonic	Mouse	IL10 KO mice and WT (C57Bl/10J)	5 × 10^6^	20	Isolate	M57-15	2, 4, 6, 8 days	Chmiel et al. (2002)
2003	Coleman	Drinking water	Planktonic	Mouse	FABP-CFTR and C57BL/6	2 × 10^7^ (mL)	NA	Both	PA14, PA01, N6, N13, FD-RD-1 (mucoid)	5–7 days (30 weeks)	Coleman et al. (2003)
2005	Bragonzi	Tracheotomized	Agar beads	Mouse	C57BL/6	1 × 10^6^ - 1 × 10^7^	50	Lab	PAO1	1, 3, 7, 14, 28 days	Bragonzi et al. (2005)
2005	Song	Tracheotomized	Alginate beads	Rat	Lewis rats	1 × 10^9^	100	Isolate	NH57388A	3, 5, 7, 10 days	Song et al. (2005)
2005	Dagenais	Tracheotomized	Agar beads	Mouse	BALB/C, DBA/2, C57Bl/6 and A/J	1 × 10^5^ to 1 × 10^6^	50	Isolate	508	1, 4, 6, 14 days	Dagenais et al. (2005)
2005	Hoffmann	Tracheotomized	Planktonic	Mouse	FABP-CFTF or BALB/c	1 × 10^8^–1 × 10^9^	40	Both	PA01, mucoid NH57388A or non-mucid NH57388B and NH57388C	13 days	Hoffmann et al. (2005)
2006	Saadane	Tracheotomized	Planktonic	Mouse	CF-KO (B6.129P2.Cftr^tmtUnc^) and WT	3 × 10^6^–4 × 10^6^	20	Isolate	PA M57-15	2, 4, 6 days	Saadane et al. (2006)
2006	van Heeckeren	Intranasal	Planktonic	Mouse	FABP-CFTR and WT	1 × 10^5^–1 × 10^9^	20	Isolate	PA M57-15	3 h–13 days	van Heeckeren et al. (2006)
2008	Kukavica-Ibruji	Intubation	Agar beads	Rat	SD	1 × 10^6^–5 × 10^6^	120	Both	PA01, PA14 and LESB58	1, 3, 7, 14 days	Kukavica-Ibrulj et al. (2008)
2009	Bragonzi	Tracheotomized	Agar beads	Mouse	C57BL/6	1 × 10^6^–2 × 10^6^	50	Both	PA01, PA14, 5 environmental strains and 25 isolates	14 days	Bragonzi et al. (2009)
2010	Rejman	Tracheotomized	Agar beads	Mouse	C57BL/6NCrlBR	2 × 10^6^	50	Isolate	RP73	14 days	Rejman et al. (2010)
2011	Growcott	Intubation	Agar beads	Rat	SD	1 × 10^5^	Not stated	Lab	PA01V	2, 5, 7 days	Growcott et al. (2011)
2011	Munder	Orotracheal	Planktonic	Mouse	FABP-CFTR, CFTR KO and WT	6 × 10^5^	30	Isolate	TBCF10839	multiple time points up to 8 days	Munder et al. (2011)
2012	Lore	Tracheotomized	Planktonic	Mouse	C57BL/6	1 × 10^5^–1 × 10^9^	50	Isolate	AA2, AA43, AA44, KK1, KK2, KK71, KK72, MF1 and MF51	12, 24 and 48 h	Lorè et al. (2012)
2012	Christophersen	Tracheotomized	Alginate beads	Mouse	BALB/c	6.6 × 10^8^–7.1 × 10^8^ (mL)	Not stated	Isolate	PA0579	1, 2, 3, 5, 6 days	Christophersen et al. (2012)
2014	Facchini	Tracheotomized	Agar beads	Mouse	C57BL/6	1 × 10^6^–5 × 10^6^	50	Both	PAO1 and RP73	3, 7, 14, 28 days	Facchini et al. (2014)
2014	Munder	Orotracheal	Planktonic	Mouse	C3H/HeN	2 × 10^6^	30	Isolate	TBCF10839lux	multiple time points up to 8 days	Munder et al. (2014)
		Orotracheal	Planktonic	Mouse		2 × 10^8^	30	Isolate	D8A6lux		
2016	Lore	Tracheotomized	Agar beads	Mouse	C57BL/6	1 × 10^6^–2 × 10^6^	50	Isolate	AA43	28 days	Lorè et al. (2016)
2016	Hengzhuang	Tracheotomized	Alginate beads	Mouse	BALB/c	1 × 10^8^	40	Both	PAO1 and NH57388A	12, 24, 72 h and 7 days	Hengzhuang et al. (2016)
		Tracheotomized	Planktonic	Mouse		5 × 10^9^	40	Both			
2016	Bayes	Tracheotomized	Agar beads	Mouse	C57BL/6	1 × 10^6^	50	Isolate	NH57388A	2 weeks	Bayes et al. (2016)
2016	Cigana	Tracheotomized	Planktonic	Mouse	C57BL/6, B6.FVB, FABP-CFTR and WT	5 × 10^6^	50	Isolate	AA2, AA43, AA44, KK1, KK2, KK71 and KK72	12 h, 1 and 2 days	Cigana et al. (2016)
			Agar beads	Mouse		1 × 10^6^–2 × 10^6^	50	Isolate		2, 14, 28 and 90 days	
2018	Cigana	Tracheotomized	Agar beads	Mouse	C57BL/6	1 × 10^6^–2 × 10^6^	50	Both	PA14, AA2, AA43	7, 13 or 21 days	Cigana et al. (2018)
2018	Lore	Tracheotomized	Planktonic	Mouse	C57BL/6	5 × 10^6^	50	Isolate	AA2	6 h	Lorè et al. (2018)
			Agar beads	Mouse		1 × 10^6^–2 × 10^6^	50	Isolate	AA43	28 days	
2019	Rosenjack	Tracheotomized	Agar beads	Mouse	CF (F508del), CF/Hdac6, Hdac6 and WT	2.5 × 10^4^	50	Isolate	PA M57-15 (mCH)	1, 3 and 5 days	Rosenjack et al. (2019)
2019	Cutone	Tracheotomized	Agar beads	Mouse	FABP-CFTR and WT	1 × 10^6^	50	Isolate	PA MDR-RP73	6 days	Cutone et al. (2019)
2020	Cigana	Tracheotomized	Planktonic	Mouse	C57BL/6NCrlBR	1 × 10^6^	50	Lab	PAO1	6 h	Cigana et al. (2020)
			Agar beads	Mouse	C57BL/6NCrlBR	5 × 10^5^	50	Isolate	MDR-RP73	7 and 14 days	
2020	Brao	Intranasal	Planktonic	Mouse	Scnn1B-Tg (B-ENaC) BALB/c and WT	1 × 10^5^–5 × 10^6^	50	Both	PA 01 and 3 isolates (non-mucoid - CF001, CF002, mucoid - CF1188)	3, 7 and 12 days	Brao et al. (2020)
2021	Cigana	Tracheotomized	Agar beads	Mouse	C57BL/6NCrlBR	3 × 10^5^ or 5.6 × 10^5^	50	Both	PAO1, RP45 and RP73	2 and 7 days	Cigana et al. (2021)
2022	Henderson	Orotracheal	Agar beads	Rat	CFTR KO (SD-CFTRtm1sage) and WT	3 × 10^6^	300	Isolate	PA M57-15	3, 7, 14 and 28 days	Henderson et al. (2022)

Several studies have investigated and compared the effect of strain on lung infection. Kukavica-Ibrugi et al*.* (2008) compared three strains of *P. aeruginosa*; two lab strains (PAO1, PA14) and a clinical isolate (LESB58) in a bead model of infection (discussed in detail below) in rats (Kukavica-Ibrulj et al., 2008). Differences were found in the *in vivo* motility and the *in vitro* biofilm formation between the three strains. The two lab strains displayed greater motility, being found inside and outside the beads, and exhibited reduced biofilm formation compared to the clinical isolate, 14 days post infection in the rat lungs. This study highlighted that all three strains were able to initiate and maintain infection in the rat lung for at least 14 days; however, the use of lab strains may not be representative of CF chronic infection.

In another study, PA14 was shown to be more virulent than the clinical isolates investigated, causing higher mortality rates in mice (Bragonzi et al., 2009). Additionally, this study compared strains isolated from CF patients with early, intermediate, and late stage lung disease, based on the number of years post *P. aeruginosa* colonisation. A higher mortality was observed in the early and intermediate isolates, suggesting there was a loss of virulence factors in late stage isolates. In a similar study, mice inoculated with early-derived *P. aeruginosa* also demonstrated higher mortality when compared to mice infected with a late-derived isolate from the same patient (Lorè et al., 2012). These studies highlight that *P. aeruginosa* clinical isolates collected from different time points post-colonisation can impact the severity of the model, and therefore is an important consideration in project planning.

To monitor the *in vivo* progression of infections typically associated with skin wound models, bioluminescent strains of *P. aeruginosa* have been developed (Thorn et al., 2007). The integration of the luxCDABE luciferase reporter gene operon into the bacteria enables luminescence detection without the addition of an exogenous substrate, allowing for real-time non-invasive quantification in skin wound models (Andreu et al., 2011). Bioluminescent *P. aeruginosa* strains have also been employed in the lungs. One study compared two strains, TBCF10839 and its isogenic less virulent mutant D8A6 (Munder et al., 2014). Mice were infected with 2 × 10^6^ and 2 × 10^8^ colony forming units (CFU) of TBCF10839*lux* or D8A6*lux* and imaged at 0, 6, and 24 h after instillation. D8A6*lux* given at the higher dose was detectable for the observation period of 24 h, whereas the more virulent *P. aeruginosa* strain TBCF10839*lux* given at higher dose resulted in early high mortality, meaning bioluminescence data could not be acquired. Bioluminescence produced by low doses of bacteria was below the limit of detection, suggesting that the option of using bioluminescent *P. aeruginosa* strains for monitoring infection progression of low-level chronic infection in the lung may be limited.

Given that there are many strains and isolates of *P. aeruginosa* that can be used in lung infection model development, researchers should determine the type of model required, then use the most suitable *P. aeruginosa* strain for their experiment. It can be argued that using a laboratory strain of *P. aeruginosa* with characteristics of being non-mucoid and highly virulent is not clinically appropriate to use as a model of the infection that persists for years in the lungs of people with CF (Hoffmann, 2007). In contrast, *P. aeruginosa* isolates that are known to be mucoid clonal strains, i.e. have a high prevalence among CF population, may produce an infection model that is more clinically relevant, and thus more useful for drug/treatment studies. It is also challenging to make direct comparisons between the characteristics of the lung infections generated by different researchers when different strains of *P. aeruginosa* are employed (as shown in Table 1). For example, those studies trialing antibiotic therapies using one strain may not translate well to different strains of *P. aeruginosa,* particularly those tested/developed using PAO1. Overall, the strain of *P. aeruginosa* used in the model can have a great impact on the outcome of the infection created, and should be chosen carefully.

#### Lung delivery methods

Multiple delivery methods exist for administering *P. aeruginosa* into the lungs of animals, and the chosen delivery technique is important as it determines the accuracy and precision of dosing, progression of infection, and has impacts on the animal’s health and welfare. Inoculation techniques include adding bacteria to the drinking water (Coleman et al., 2003), intranasal application (Schroeder et al., 2001; van Heeckeren et al., 2006; Brao et al., 2020), tracheostomy (van Heeckeren et al., 2000; Hoffmann et al., 2005; Bayes et al., 2016) and non-surgical intratracheal delivery (Schroeder et al., 2001; Munder et al., 2011) (Table 1). Some factors that should be considered when deciding on which technique to use to deliver *P. aeruginosa* to the lungs include the target location and the number of bacteria to be delivered to that location, minimising contamination of non-target tissue, the reproducibility of the technique, and the physiological impact on the animal (e.g. choice of anaesthetic and invasiveness of the delivery procedure) (Munder et al., 2002).

In efforts to replicate environmental low-level transmission of *P. aeruginosa* to the lung, one study showed that long term colonisation after exposure to *P. aeruginosa* in drinking water in wildtype (WT) and CF mice mimics early CF lung disease (Coleman et al., 2003). *P. aeruginosa* colonised the oropharyngeal cavity with natural progression to the lower airways after long term colonisation. Further, this study reported that WT mice were able to clear the infection once the *P. aeruginosa* water source was removed, whereas CF mice did not, indicating differences between the WT and CF mice susceptibility to *P. aeruginosa* infection (Coleman et al., 2003).

As sinuses can be a source of lung infection (Chaaban et al., 2013), another method that may mimic a natural acquisition of bacteria to the lungs is intranasal delivery to simultaneously target both upper and lower airways. This involves the mouse being held in a vertical position or laying on its side to deliver small volumes of the *P. aeruginosa* solution to the tip of the nose, allowing the animal to passively inhale the bacteria. Further, the use of light anaesthesia minimises the amount of bacteria that might otherwise get swallowed if the animal was awake (Bakker-Woudenberg, 2003). To be able to develop a chronic and established infection with this method, multiple inoculations would be required, as only small numbers of bacteria reach the lung.

The most widely used method of delivering *P. aeruginosa* to the lungs is tracheostomy, especially in mice. This approach is typically chosen due to the accuracy of delivery as the bacteria are directly inoculated to the lung. However, this method is complex, invasive and requires a high skill level to surgically expose the trachea and close the wound postoperatively. Further, it brings with it the potentially confounding effect of delayed recovery as a result of the surgery as well as increased procedure-associated mortality. Delivery factors that may contribute to this associated mortality include airway obstruction due to the use of a large gauge blunt (28% increase in mortality) and inappropriate placement of the angiocatheter (60% increase in mortality) (van Heeckeren and Schluchter, 2002). A separate study reported that from 96 mice undergoing infection *via* tracheostomy, six died due to postoperative complications and seven were improperly infected (Van Heeckeren et al., 2000). Consequently, the limitations of delivery by tracheostomy likely outweigh the benefits, particularly when other more accurate and lower-impact delivery methods exist. Additionally, some animal ethics committees may be reluctant to approve this method of delivery.

To avoid the need for tracheostomy, *P. aeruginosa* can instead be delivered to the lungs through an endotracheal (ET) tube or cannula that is inserted into the trachea *via* the mouth (MacDonald et al., 2009). Although minimally-invasive, a high skill level is required for this method. The ET tube is easily inserted into the oesophagus, resulting in erroneous delivery to the stomach. Confirmation of ET placement can be achieved by using a puff of air to inflate the lungs and cause the chest movement. Whole lung delivery is achieved if the ET tube is inserted correctly into the trachea, whereas some studies report that it is possible to achieve half lung delivery by angling the ET tube to one side, (e.g. right main bronchus) (Saadane et al., 2006).

Munder et al. (2011) uses a method called “view controlled intratracheal infection” (orotracheal), which consists of anaesthetising the mice, stretching the tongue out and visualising the instillation of bacteria directly into trachea using a magnifying glass or a small animal laryngoscope (Munder et al., 2011). This method differs from above as no ET tube is inserted, and the inocula is instead delivered straight to the top of the trachea. In another study, the same group compared several methods of delivery, concluding that this method was the preferred approach due to accuracy of dosing and recovery of mice (Munder et al., 2002). In their study, tracheostomy resulted in the highest numbers of bacteria in the lung, but was the only method in which the delivery procedure was associated with mortality. The least invasive methods, which might be suitable for simulating environmental exposure to *P. aeruginosa* - intranasal and aerosol delivery - resulted in low level bacterial colonisation in the lung, poor reproducibility, and contamination of other tissues.

Each delivery method has advantages and disadvantages that must be taken into consideration. Ideally, a lung infection model should simulate the human route of infection as closely as possible. Some of these methods (i.e. intranasal) may be less reproducible between experiments, however those methods able to deliver the same amount of bacteria each time to the lung creates a model that has lower variation between the groups of animals. Therefore, having a delivery method that limits dosing to a specific region, is reproducible and minimally invasive would be advantageous. A newly established method that employs miniature bronchoscope fits this need, and can enable a more accurate and precise method for localised fluid delivery to the lungs, particularly in large lab animals such as rats (McIntyre et al., 2018). As this procedure can be performed rapidly (∼1 min), it is achievable, using inhaled anaesthesia, and therefore has the added benefit of reducing complications associated with surgical methods and injectable anaesthetics.

#### Duration of infection

In humans with CF, *P. aeruginosa* infection begins with recurrent acute colonisation of the airways, before developing into a long-lasting chronic infection, typically after or in parallel with infection with other pathogens. Approaches to inducing appropriate lung infections have typically been designed to produce either short-term (acute) or persistent long-term (chronic) infections in animal models (Table 1).

#### Short-term (acute) infection

The administration of planktonic (free-living) *P. aeruginosa* is a method used to create a short-term model of lung infection. However, depending on the number of CFU used this approach can result in the two extremes of rapid clearance without inducing infection, or acute sepsis and death. For example, mice (CF—FABPCFTR and WT) that were intranasally inoculated with a high number of bacterial CFU (10^9^) died within 2 days, whereas those with a lower CFU (10^5^) cleared the infection rapidly (van Heeckeren et al., 2006). The authors concluded that an intermediate dose of 10^7^ CFU, was associated with weight loss but no mortality and thus used this dose in subsequent experiments. Similarly, Saadane et al. (2006) also concluded that administration of 3–4 x 10^6^ CFU resulted in improved survival when compared to using a higher dose of bacteria in CF knockout (KO) and WT mice (Saadane et al., 2006).

The single dose planktonic delivery approach in rodents does not and cannot reflect the chronic lung infection that persists in people with CF. However, this delivery approach can be appropriate for investigating some aspects of lung infection, such as the acute initial inflammatory response following lung infection (Cigana et al., 2020), comparing lab strains to isolates of *P. aeruginosa,* assessing the response of infection in different strains of rodents, and evaluating treatments that could modify the course of early infection (e.g. antibiotics).

#### Long-term (chronic) infection

The definition of a chronic lung infection in humans with CF is varied, but two common definitions are well accepted: 1) The ‘Leeds criteria’, which refers to the isolation of *P. aeruginosa* in >50% of sputum cultures over a 12-month period, and, 2) at least three positive cultures for >6 months, with a minimum 1-month interval between samples (Lee et al., 2003; Proesmans et al., 2006). However, these durations represent a much greater proportion of an animal’s lifespan, and equivalent definitions are therefore required for application to animal models.

Cigana et al. (2016) suggested recovery of more than 10^4^ CFU of bacteria in the bronchoalveolar lavage fluid (BALF) of mice 3 months after inoculation to be indicative of a chronic infection (Cigana et al., 2016). In that study, hallmarks of chronic lung pathology were displayed 1-month post-infection, including intraluminal and peribronchial inflammation, epithelial hyperplasia and structural degeneration. This study was the first to develop long-term chronic infection with stable bacteria numbers persisting for up to 3 months. Bayes et al. (2016) developed a chronic infection in mice that demonstrated key features of CF human lung disease, with a low level of morbidity, neutrophilic inflammation and weight loss related to infection (Bayes et al., 2016). Chronic infection in this study was defined as the recovery of *P. aeruginosa* from BALF and homogenised lung cultures 2 weeks post inoculation with a median count of 1.4 × 10^3^ CFU/lung. Similar, Bragonzi et al. (2009) and Lore et al. (2016) used the recovery of more than 10^3^ CFU in whole lung cultures as an indication of chronic lung disease (Bragonzi et al., 2009; Lorè et al., 2016). Ultimately, it is important to select a bacterial load that maintains a stable infection without significantly compromising the health of the animal.

#### Creating a chronic infection model

In humans with CF, a chronic *P. aureuginosa* lung infection is maintained at a low level with the aid of a polysaccharide matrix generated by the bacteria, which retains the bacteria in the lung. To attempt to replicate the chronic infection in animal models, *P. aeruginosa* is often embedded into agar or alginate beads, which helps to physically retain the bacteria in the airways and delay the clearance of infection. These agents also prevent neutrophils accessing the bacteria, thus neutrophils remain in the airways as is seen in CF (Chmiel et al., 2002). This technique is designed to generate persistent presence of bacteria in the lungs, where it would cause continuous stimulation of host defences and inflammation (Growcott et al., 2011; Facchini et al., 2014).

Cash et al*.* (1979) first devised the *P. aeruginosa* agar embedded bead model of chronic infection in rats (Cash et al., 1979), and it has since been modified for other species (Thomassen et al., 1984; Cheung et al., 1993; Collie et al., 2013), such as mice (Starke et al., 1987). Agar beads are commonly made by adding *P. aeruginosa* culture into warmed agarose and then mixing it into warmed mineral oil to form beads. An alternative bead model using alginate was developed by Pedersen et al. (1990), as the *P. aeruginosa* biofilms in humans with CF consist of an alginate matrix containing the bacteria (Pedersen et al., 1990). Alginate beads are generally made by forcing a bacteria laden alginate mixture through a needle into a stirred calcium chloride solution (Delbari et al., 2014).

Agar and alginate are both polymers derived from seaweeds, but they have different chemical properties (Torres et al., 2019). Agar is a neutrally-charged polysaccharide while alginate is anionic, which means they interact with antibiotics differently. The choice of which substance to use for bead production will therefore depend on the nature of the study. Calcium-alginate and agar beads have been compared in an *in vitro* study testing antibiotic therapies against *P. aeruginosa* (Torres et al., 2019). This study concluded that these two substances are interchangeable if evaluating antibiotic therapy with uncharged antibiotics. However, testing the efficacy of positively charged antibiotics requires the use of calcium-alginate beads to enable the antibiotic to reach the bacteria.

Bead size is variable depending on the method, equipment, and the operator. Importantly, beads must not be so large that they cause obstruction in large conducting airways. One study used different sizes of alginate beads to investigate the localisation of infection in mice: smaller beads were between 15 and 85 μm and large beads 74–205 μm (Christophersen et al., 2012). Distinct inflammatory responses within differing pulmonary zones were observed in mice that received the smaller beads compared to those that received the larger beads. Importantly, studies have shown that although sterile beads create an inflammatory response when delivered to the lungs, a greater inflammatory response and histological changes occur in those that receive the bacterial laden beads (Bayes et al., 2016).

Producing beads of similar distribution and size within and between batches can be technically demanding. One study found that using non-ionic surfactant SPAN 80 in the agar bead production method ensured reproducible production of homogeneous bead preparations (Growcott et al., 2011). Other factors that affect the agar bead size include the rate of stirring and cooling of the mineral oil, with higher speeds decreasing the size of beads and slower speeds producing larger beads, and the different operators who are making the agar beads (van Heeckeren and Schluchter, 2002; Facchini et al., 2014). Alginate bead size can be altered somewhat by changing the gauge of the needle the alginate mixture is passed through, and by varying the pressure/flow rate used.

Despite using standardised production methods, the properties of each bead batch are typically highly variable, resulting in different size distributions, total numbers of beads, and numbers of bacteria in each bead. With each inoculate of *P. aeruginosa* beads delivered to the animal, how does the researcher know how many beads are being delivered? Each batch must be validated with CFU counts and appropriate numbers of animals used to overcome the production variability. Studies also tend to report how many bead batches were used in animal experiments to account for potential discrepancies arising from bead lot, but interestingly researchers rarely report the number of beads delivered even though this is likely to have a major impact on the level of infection. Despite the difficulties in making and using beads, these models remain widely used because they can successfully establish a chronic infection.

These chronic bead models are typically more relevant for investigating CF lung disease. The benefits of embedding *P. aeruginosa* into beads include preventing bacterial clearance, slowing the growth of the bacteria, and avoiding the acute sepsis that is a risk of the planktonic model. The persistence of the *P. aeruginosa* infection in the lungs of these animal models reproduces much of the reported CF lung pathology including lung histopathology, increased inflammatory and cytokines responses. These models have been used in studies of CF lung disease and bacterial pathogenesis (Dagenais et al., 2005; Kukavica-Ibrulj et al., 2008; Bruscia et al., 2016) and in treatment studies, such as those using antibiotics or gene therapy (Rejman et al., 2010; Growcott et al., 2011; Lorè et al., 2018; Cigana et al., 2021).

#### Selection of rodent strain

Several studies have compared CF and WT animal responses to *P. aeruginosa* infection. Van Heekeren et al. (2006) reported that CF KO mice have higher mortality than WT mice at high doses of *P. aeruginosa*, whereas at lower doses CF mice survive but with a greater weight loss than WT mice (van Heeckeren et al., 2006). In lower dose studies the CF mice are able to clear the bacteria just as quickly as WT mice, suggesting that the increased weight loss is due to the exaggerated inflammatory response induced by the infection. In another study, an elevated inflammatory response was observed in CF mice during the early time points compared to the WT mice (Cigana et al., 2016). Chronic infection differed histologically between the CF and WT mice, with a higher number of goblet cells, collagen deposition, and elastin degradation observed in the CF lungs. Their findings suggest that the inflammatory response and tissue damage created during *P. aeruginosa* infection is dependent on the presence of functional CFTR in mouse models, similar to the situation in people with CF (Cigana et al., 2016).

Recently, Henderson et al. (2022) compared the development of a chronic *P. aeruginosa* infection in younger (2-month-old) and older (6-month-old) CFTR KO rats using an agar bead method (Henderson et al., 2022). Age matched WT rats were also included in the study. The authors found that the 2-month-old KO rats demonstrated a similar course of infection to their age-matched WT rats, where the bacteria was cleared within 7 days of infection and all other indications of infection had returned to baseline. The 6-month-old KO rats on the other hand, indicated an inability to clear the infection, and showed increased inflammation and mucus accumulation when compared to the WT rats of the same age. The authors concluded that the older KO rats likely developed chronic infection due to the presence of hyperviscous and static airway mucus presence, which was not present in the younger KO or older WT rats (Henderson et al., 2022).

*Scnn1b*-transgenic mice, a model of CF-like lung disease mice, have been used in bacterial studies. These mice overexpress the β subunit of the epithelial sodium channel (β-ENaC) in the airway epithelium, resulting in increased sodium absorption similar to CF. In both studies these β-ENaC mice cleared the infections more slowly than the WT mice, indicating impaired clearance (Mall et al., 2004; Brao et al., 2020). One of the studies further investigated the effect of PAO1 and non-mucoid or mucoid CF clinical isolates in cytokine responses and histological features (Brao et al., 2020). It was concluded that this mouse strain modeled early *P. aeruginosa* colonisation when a muciod strain was delivered modelled early *P. aeruginosa* colonisation. A limitation here was that most of the β-ENac mice cleared the bacteria by day 12, perhaps due to the planktonic intranasal method used.

Immunocompromised mice (genetic mouse strains or induced *via* immunosuppressants) have also been employed for modelling lung infections. One study found that mice who were made immunocompromised by treatment with cyclophosphamide or neutrophil-depleting RB6-8C5 monoclonal antibody were hypersusceptible to *P. aeruginosa* infection, compared to immunocompetent/healthy animals (Scarff and Goldberg, 2008). Lawrenz et al. (2015) demonstrated that immunocompromised animals may be useful as they are much more susceptible to infection, but the risk of morbidity and mortality is greater in these models (Lawrenz et al., 2015). While these models may have utility for particular research applications (e.g. (Chang et al., 2022)), the absence of a functional immune system may limit the translation of study findings to humans.

There are a range of rodent strains that have been used for *P. aeruginosa* lung infection models, and each has factors to consider when selecting the appropriate strain (Table 1). CF rodents and β-ENaC mice showed differences in their response to the infection, when compared to WT counterparts, and are valuable in the understanding of CF pathogenesis in response to infection. However, CF strains may not be essential in the initial steps of treatment-development studies targeting the bacteria. Other considerations when deciding to use non-CF or CF animals are the availability, cost, husbandry, and production levels that are an inherent part of CF breeding.

Ultimately, there are advantages and disadvantages to the different experimental designs described for generating a *P. aeruginosa* lung infection model. Table 2 summaries the pros and cons of these strategies to aid researchers in selecting the most appropriate approach for their investigation.

**TABLE 2:** Summary of experimental design pros and cons for developing a *Pseudomonas aeruginosa* infection model.

Experimental design		Pro	Con
*P. aeruginosa* strain	Laboratory	Early course of infection studies	Highly virulent
			Non-mucoid
			May not recapitulate antibiotic outcomes
	Clinical Isolate	Biofilm formation	Need to determine if using an early, intermediate or late stage *P. aeruginosa* strain
		Mucoid variants available	
		Recapitulate antibiotic outcomes	
		Clinically relevant	
Lung delivery method	Tracheotomized	100% of inoculate to lungs	High technical skill level is required
		High reproducibility	Surgery required
			Associated procedure mortality
	Intranasal	Easy to perform and non-invasive	Bacterial load delivered to lungs may not be sufficient to generate infection
		Mimics natural acquisition of bacteria to lung	High variability in bacterial load delivered to lung
			High numbers of animals needed to overcome variability
			Possible that bacteria are swallowed
			Multiple inoculations required for chronic infection
	Orotracheal	Less invasive	Difficult to know if inoculum went into lungs or stomach
	Drinking water	Replicates environmental low-level transmission	Long-term study needed to accumulate bacteria in lungs
			Possible contamination of non-target tissue
			Non-invasive
			Unknown amounts of bacteria delivered to lung
			High variability, high numbers of animals needed
	Intubation	Less invasive	High technical skill level is required
		Low variability, therefore less animals needed	
		Can blindly deliver tone side of the lung if required	
		Reproducible	
	Bronchoscope	Low variability, therefore less animals needed	High technical skill level is required
		Precise delivery to the lung	
		Can precisely deliver to a single lobe if required	
		Reproducible	
State of *P. aeruginosa*	Planktonic	Useful in early course of infection studies	Rapidly cleared
		Useful in studies comparing *P. aeruginosa* strains	If dose is too high acute sepsis or death
		Investigating inflammatory responses	Does not reflect CF
	Embedded in beads	Retains the bacteria in airways longer	Technically demanding
		Delays clearance	Bead batches highly variable
		Avoids acute sepsis and death	Size of beads is important
		Persistent infection	Bead model artificial way of producing chronic infection
Rodent strain	WT	Useful in initial steps of developing infection studies	Does not replicate the histopathology seen in CF lung disease
		Highly available	Different inflammation responses to humans with CF
		Cheaper to use	
	CF	Histologically relevant infection	Increased husbandry demands
		Similar inflammation responses to humans with CF	Not readily available
			Higher costs
			Reduced breeding capacity
	Immunocompromised	Susceptible to *P. aeruginosa* infection	Greater risk of mortality
			Absence of functional immune system
			Does not produce CF lung disease

### Limitations of induced lung infection models

Although *P. aeruginosa* lung infections in animal models are useful for the study of CF lung disease, they are not free of limitations. People with CF typically have progressive lung disease caused by colonisation of a variety of bacterial species that changes over time in response to a range of factors. Therefore, the direct delivery of *P. aeruginosa* into the sterile lungs does not mimic this natural progression whereby certain bacterial species become dominant in CF lung infections. For accurate representation, where appropriate, studies should consider co-infection as well as sequential infection models (Millette et al., 2019).

Models that use high doses of a laboratory strain of *P. aeruginosa*, resulting in acute sepsis and mortality, do not recapitulate typical features of CF lung disease in particular the steady low-level introduction of *P. aeruginosa* from the normal daily environment. Further, some CF animal models appear to be able to clear *P. aeruginosa* without the aid of a substance to retain it in the lung, whereas people with CF are unable to clear the bacteria. However, this may be due to those CF animals falling to develop hallmark features of CF lung disease, such as mucus obstruction, that prevent clearing of the bacteria.

### Concluding remarks

As all current CF rodent models do not develop spontaneous or chronic lung disease, efforts to induce a more CF-like lung disease phenotype have been pursued. Taken together, the literature demonstrates that the development of a *P. aeruginosa* infection model for CF can have varied success, and establishing the correct method and dose for a given experimental question about CF-like lung infection can be challenging. There are many aspects to be considered, including the strain of *P. aeruginosa*, method of *P. aeruginosa* delivery and if planktonic forms are not suitable, which is the optimal substance for *P. aeruginosa* to be delivered within. Nonetheless, selecting the right model for the research application is important so that the most informative and reliable outcomes can be obtained. Additionally, future implementation of a standardized reporting method in the literature would be beneficial to further aid researchers in comparing and selecting the most suitable experimental approach for their study. In conclusion, no single rodent model fully recapitulates all aspects of CF lung disease, however these infection models are undoubtedly essential tools for developing novel therapeutic strategies and improving understanding of CF pathogenesis and mechanisms to progress to the clinic.
